# Response of plasmaspheric configuration to substorms revealed by Chang’e 3

**DOI:** 10.1038/srep32362

**Published:** 2016-08-31

**Authors:** Han He, Chao Shen, Huaning Wang, Xiaoxin Zhang, Bo Chen, Jun Yan, Yongliao Zou, Anders M. Jorgensen, Fei He, Yan Yan, Xiaoshuai Zhu, Ya Huang, Ronglan Xu

**Affiliations:** 1 National Astronomical Observatories, Chinese Academy of Sciences, Beijing, China; 2 Key Laboratory of Solar Activity, Chinese Academy of Sciences, Beijing, China; 3 School of Natural Sciences and Humanity, Harbin Institute of Technology Shenzhen Graduate School, Shenzhen, China; 4 State Key Laboratory of Space Weather and National Space Science Center, Chinese Academy of Sciences, Beijing, China; 5 National Center for Space Weather, China Meteorological Administration, Beijing, China; 6 Changchun Institute of Optics, Fine Mechanics and Physics, Chinese Academy of Sciences, Changchun, China; 7 Electrical Engineering Department, New Mexico Institute of Mining and Technology, Socorro, New Mexico, USA

## Abstract

The Moon-based Extreme Ultraviolet Camera (EUVC) of the Chang’e 3 mission provides a global and instantaneous meridian view (side view) of the Earth’s plasmasphere. The plasmasphere is one inner component of the whole magnetosphere, and the configuration of the plasmasphere is sensitive to magnetospheric activity (storms and substorms). However, the response of the plasmaspheric configuration to substorms is only partially understood, and the EUVC observations provide a good opportunity to investigate this issue. By reconstructing the global plasmaspheric configuration based on the EUVC images observed during 20–22 April 2014, we show that in the observing period, the plasmasphere had three bulges which were located at different geomagnetic longitudes. The inferred midnight transit times of the three bulges, using the rotation rate of the Earth, coincide with the expansion phase of three substorms, which implies a causal relationship between the substorms and the formation of the three bulges on the plasmasphere. Instead of leading to plasmaspheric erosion as geomagnetic storms do, substorms initiated on the nightside of the Earth cause local inflation of the plasmasphere in the midnight region.

The plasmasphere contains dense and cold thermal plasmas that surround and corotate with the Earth^1^. The plasmas in the plasmasphere originate from the ionosphere and are trapped by the magnetic field of the Earth^1^. In empirical and theoretical models^2^,^3^,^4^,^5^,^6^,^7^,^8^,^9^, the plasmasphere is in a torus structure since the filling of the ionospheric plasmas to the plasmasphere is along the geomagnetic dipole field lines^10^, and thus the outer boundary of the plasmasphere (called plasmapause, where the plasma density drops by 1–2 order of magnitude^11^) can be fitted with the dipole field model of the Earth^4^,^8^. The plasmasphere is one inner component of the whole magnetosphere and its configuration is sensitive to magnetospheric activity (storms and substorms)^1^,^12^. Empirically, the size of the plasmasphere is related to the planetary geomagnetic disturbance level scaled by the *Kp* index^2^,^4^. The typical radial extension distance of the plasmapause is 4–6 Earth radii (*R*_E_) for relatively quiet geomagnetic condition^2^, while during severe magnetic storms, the plasmasphere can shrink significantly as the result of the erosion process in the inner magnetosphere^1^,^12^. Substorms initiated on the nightside of the Earth^13^ may also affect the configuration of the plasmasphere^1^,^5^,^11^,^12^,^14^,^15^,^16^,^17^, but the physical process is only partially understood in observation and theory^11^.

In addition to *in situ* measurement of plasma density via spacecraft, the configuration of the plasmasphere can be observed by remote imaging^18^. Large field of view (FOV) imaging of the plasmasphere at 30.4 nm Extreme Ultraviolet (EUV) wavelength (sunlight spectral line resonantly scattered by the He^+^ ions in the plasmasphere)^18^ can reveal the global plasmaspheric configuration, the fine structure of the plasmasphere, and their time evolution^19^,^20^, as demonstrated by the data from the EUV Imager^21^,^22^ aboard the IMAGE (Imager for Magnetopause-to-Aurora Global Exploration) satellite^23^,^24^ with an equatorial view (top view)^19^ of the plasmasphere, and the Telescope of Extreme Ultraviolet (TEX) aboard the lunar orbiter KAGUYA with a meridian view^25^,^26^,^27^,^28^. The Extreme Ultraviolet Camera (EUVC)^29^ carried by the lunar lander of the Chang’e 3 mission^30^ is the latest instrument working at the 30.4 nm wavelength band for remote imaging observation of the plasmasphere (see Methods for the EUVC imaging principle). Because the EUVC is fixed on the lander of Chang’e 3, the track of the EUVC in space is basically the same as the orbit of the Moon, which is roughly 60 *R*_E_ away from the Earth. Thus it can provide a global and instantaneous meridian view (side view)^31^,^32^ of the Earth’s plasmasphere and its observations provide a good opportunity to investigate the responses of the plasmaspheric configuration to substorms. Comparisons with simultaneous *in situ* data from various space missions have demonstrated the reliability of the EUVC data for identifying plasmapause locations^33^,^34^,^35^.

Here we use the EUVC data obtained on 20–22 April 2014 to analyze the shape of the plasmaspheric configuration and its relationship with substorms (see Methods for the EUVC data reduction procedure). The cadence of the EUVC images is about 10 minutes^31^, and the time span of the successively observed images is about 28 hours (from 21:02 UT on 20 April 2014 to 01:10 UT on 22 April 2014)^32^, which covers more than one Earth’s rotation. There are 153 frames of observed images in total^32^. This time series of EUVC images demonstrates the change of the profiles’ shape of the plasmasphere when the whole plasmasphere corotates with the Earth (see Supplementary Video 1 for an animation of the 153 EUVC images). By reconstructing the global plasmaspheric configuration based on these EUVC images, we find that in the observing period, the plasmasphere had three bulges which were located at different geomagnetic longitudes. The inferred midnight transit times of the three bulges, using the rotation rate of the Earth, coincide with the expansion phase^36^ of three contemporarily happened substorms. This result implies a causal relationship between the substorms and the formation of the three bulges on the plasmasphere.

## Results

### Meridian view of the plasmasphere seen by EUVC

**Figure 1:**
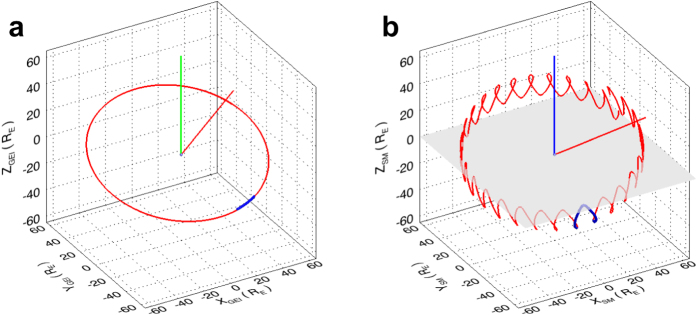
Tracks of the EUVC during the observing period. (**a**) Track of the EUVC in GEI coordinate system. The track of the EUVC during the observing period (from 21:02 UT on 20 April 2014 to 01:10 UT on 22 April 2014) is shown by the short blue curve. The red curve is the orbit of the Moon in the whole month of April 2014. The green bar indicates the rotation axis of the Earth (northern part) and the red bar gives the direction of the Sun at 12:00 UT on 21 April 2014. (**b**) Track of the EUVC in SM coordinate system. The blue bar indicates the geomagnetic dipole axis (northern part), and the shading horizontal surface represents the magnetic equatorial plane.

In Fig. 1, we show the track of the EUVC in space during the observing period of the EUVC images (short blue curve). The orbit of the Moon in the whole month of April 2014 is also displayed in Fig. 1 for reference (red curve). Figure 1a utilizes the Geocentric Equatorial Inertial (GEI) coordinate system and Fig. 1b illustrates the EUVC track and Moon orbit in Solar Magnetic (SM) coordinate system (see Methods for the definitions of the GEI and SM coordinate systems). It can be seen in Fig. 1 that during the observing period, the EUVC is roughly located in the dawn sector of magnetic local time (MLT), thus both the dayside and the nightside plasmasphere can be seen by the EUVC simultaneously.

**Figure 2:**
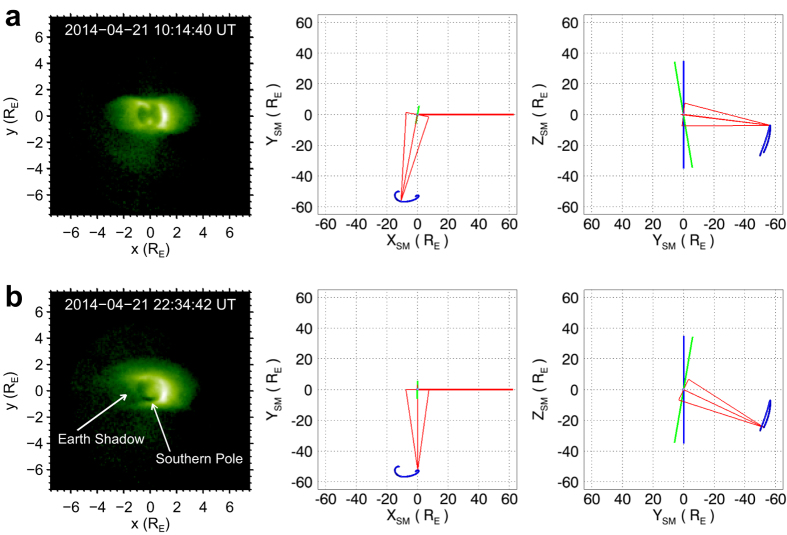
EUVC images observed from two representative viewpoints. (**a**) EUVC image (left column; log scaled image) of the plasmasphere obtained at 10:14 UT on 21 April 2014 when the EUVC was approaching the location with the minimum angle (6.9°) to the magnetic equatorial plane on 21 April 2014. The x-axis of the image is parallel to the magnetic equatorial plane and the y-axis is parallel to the projected direction of the magnetic dipole axis. The diagrams in the middle and right columns illustrate the viewpoint of the EUVC in SM coordinates when observing the image. The short blue curve is the track of the EUVC. The blue bar represents the magnetic dipole axis, the green bar represents the Earth’s rotation axis, and the red bar represents the projected direction of the Sun. The three thin red lines originated from the EUVC location in each diagram indicate the line of sight (LOS) direction and FOV of the EUVC image. (**b**) EUVC image of the plasmasphere obtained at 22:34 UT on 21 April 2014 when the EUVC was approaching the location with the maximum angle (25.2°) to the magnetic equatorial plane on 21 April 2014. The shadow of the Earth and the southern pole of the plasmasphere can be seen in the image owing to the larger angle of the viewpoint to the magnetic equatorial plane (Z_SM_ = 0).

When discussing configurations of the plasmasphere, we are more interested in the magnetic coordinate systems (such as the SM coordinate system used in Fig. 1b), since the plasmasphere is confined by the geomagnetic field^1^. From Fig. 1b it can be found that during the observing period (20–22 April 2014), the EUVC was not exactly located on the magnetic equatorial plane (indicated by a shading horizontal surface in Fig. 1b), but was located in the south of the magnetic equatorial plane. Thus the scene captured by the EUVC during the observing period is not an exact meridian view, but a quasi-meridian view of the plasmasphere. In the left column of Fig. 2, we give two pictures of the plasmasphere obtained from two representative viewpoints of the EUVC. The two images were taken at 10:14 UT and 22:34 UT on 21 April 2014, respectively, when the EUVC was approaching the locations with the minimum angle (6.9°) and maximum angle (25.2°) to the magnetic equatorial plane on 21 April 2014 (see the middle and right columns of Fig. 2). Figure 2 demonstrates different projected shapes of the torus plasmasphere as seen from different viewpoints along the rolling track of the EUVC in the SM coordinate system. Particularly, in Fig. 2b (with the larger southward angle of the viewpoint to the magnetic equatorial plane), the southern pole of the plasmasphere (and also the southern pole of the geomagnetic field) is exposed to the observer and can be identified as a dark hole in the EUV image owing to the absence of plasmas.

### Plasmaspheric configuration reconstructed from the EUVC images

**Figure 3:**
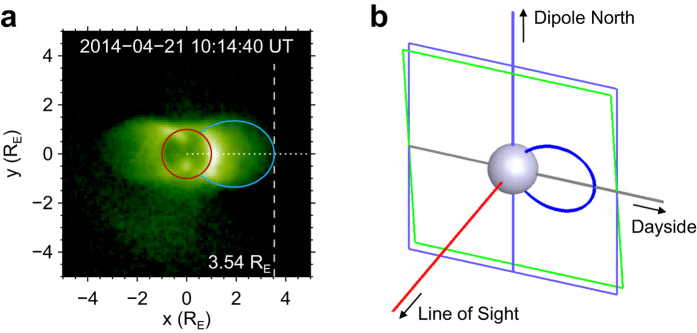
Plasmapause shape profile fitted with a selected dayside geomagnetic dipole field line. (**a**) An example EUVC image observed at 10:14 UT on 21 April 2014 (the same image as in Fig. 2a). The shape of the Earth^32^,^44^ is outlined by a red circle. The blue curve is the projection of the selected fitting field line for the dayside plasmapause shape profile. The horizontal dotted line indicates the intersection of the magnetic equatorial plane with the image plane. The location of the vertical dashed line indicates the radial distance of the field line at the magnetic equatorial plane, whose exact value (*L*-value) is 3.54 *R*_E_. (**b**) Diagram illustrating the relative positions of the EUVC image plane (green frame), the selected dayside dipole field line (blue curve), and the meridian plane (blue frame) containing the dipole field line. The central blue sphere in the diagram represents the Earth. The intersection between the image plane and the meridian plan is indicated by a gray horizontal line, which coincides with the horizontal dotted line in panel a.

To reconstruct the global plasmaspheric configuration from the EUVC images, we first fit the shape profile of the dayside plasmapause using field lines in the geomagnetic dipole field model. We are concerned with the dayside plasmapause because it is more distinct without the shadow of the Earth. For each of the EUVC images, we select a dayside dipole field line in the geomagnetic meridian plane close to the EUVC image plane to fit plasmapause boundary. This meridian plane should be vertical to the plane determined by the line of sight (LOS) direction and the magnetic dipole axis. The plasmapause boundary in the image for field line fitting is identified via visual inspection, in which we use a threshold of intensity (28 counts per pixel, see Methods for the physical meaning of image intensity) to get a rough dayside plasmapause position for reference. Figure 3 illustrates an example of a dayside plasmapause shape profile fitted with the geomagnetic dipole field line based on the EUVC image observed at 10:14 UT on 21 April 2014 (the same image as in Fig. 2a). Figure 3a shows the image with the selected dayside dipole field line (blue curve) projected on the image plane. Figure 3b demonstrates the relative position between the image plane (green frame) and the meridian plane (blue frame) that contains the selected dipole field line.

**Figure 4:**
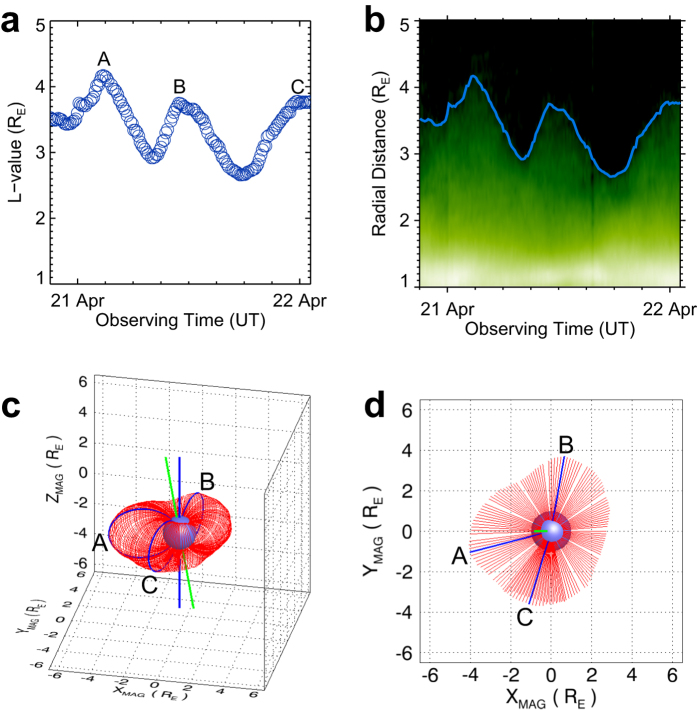
Determined plasmapause positions and reconstructed plasmaspheric configuration with the dayside fitting field lines. (**a**) Curve of the *L*-values with observing times for the 153 fitting field lines. Each circle symbol has its corresponding field line. The radius of the circle symbols (according to the vertical axis of the plot) is 0.1 *R*_E_ which is the pixel resolution of the EUVC data (see Methods) and represents the uncertainty in the determined plasmapause positions. The three peaks of the curve are labeled A, B, and C, respectively. (**b**) Time-distance diagram illustrating the dayside radial intensity distribution variation with the observational time of the 153 EUVC images. The curve of *L*-values is plotted overlying the diagram for comparison. (**c**) Three-dimensional view of the combined 153 fitting field lines (red color) in MAG coordinate system. The three bulges labeled A, B, and C correspond to the three peaks of the *L*-value curve in panel a. The field lines that have the largest radial distances in each of the bulges are highlighted in blue color. The blue bar indicates the geomagnetic dipole axis and the green bar indicates the rotation axis of the Earth. (**d**) Top view of the combined field lines. Some field line positions are void owing to the absence of corresponding EUVC imaging data. There exist overlapping field lines because the time span of the employed EUVC data covers more than one Earth’s rotation.

After performing the dayside plasmapause profile fitting for the 153 EUVC images, we have the coordinates of 153 field lines. The radial distances of the field lines at the magnetic equatorial plane are quantified by their *L*-values. The curve of the *L*-values with observing times is plotted in Fig. 4a. Three peaks of the *L*-value curve can be identified in Fig. 4a, which are labeled using the capital letters A, B, and C, respectively. We also extracted the dayside radial intensity distributions along the horizontal dotted line in Fig. 3a for all the EUVC images and obtained the time-distance diagram of the radial intensity, which is presented and compared with the *L*-value curve in Fig. 4b. It can be seen in Fig. 4b that the *L*-values determined from the profile fitting process are consistent with the variation of plasmapause locations illustrated in the time-distance diagram.

We employ the geomagnetic (MAG) coordinate system (see Methods for the definition of the MAG coordinate system) to reconstruct the global plasmaspheric configuration based on the 153 field lines. It is widely accepted that the plasmaspheric plasmas are confined in the field lines and corotate with the Earth^1^. In the MAG coordinate system, the corotation effect is eliminated and the plasmasphere can be considered as stationary (i.e., no rotation), which is convenient for the analysis of the global configuration. Because MAG longitude value of dayside direction changes over time, each of the 153 dipole field lines is given a corresponding MAG longitude. By combining all the field lines together in the MAG coordinate system, we obtain the global configuration of the plasmasphere during the observing period. The 3D view and top view of the reconstructed plasmaspheric configuration are shown in Fig. 4c,d. It can be seen from Fig. 4c,d that, during the observing period, the plasmasphere had three bulges (labeled A, B, and C, respectively) located at different geomagnetic longitudes, which correspond to the three peaks of the *L*-value curve in Fig. 4a.

### Verification of the plasmaspheric configuration with *in situ* measurements

**Figure 5:**
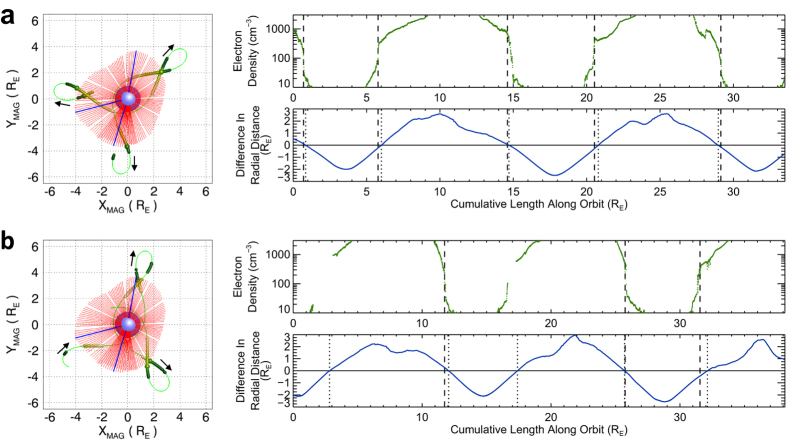
Comparison between the reconstructed plasmaspheric configuration and the *in situ* electron density data of VAP. (**a**) Comparison with the electron density data of the VAP-A satellite. The left column illustrates the orbit of VAP-A (thin green line) from 00:00 UT to 24:00UT on 21 April 2014 in the MAG coordinate system overlying the reconstructed plasmaspheric configuration (red color). The small arrows indicate the moving direction of the satellite along the orbit. The thick green lines indicate the segments of orbit with valid electron density measurements (darker color representing lower density value). The plasmapause locations determined from the electron density data are indicated by the enlarged spherical symbols on the orbit. The right column shows the distribution of the *in situ* electron density values along the orbit of VAP-A (upper plot; gaps in the curve correspond to unreliable or missing data) and the difference in the radial distance between the VAP-A location and the plasmapause surface of the reconstructed configuration (lower plot; the value being negative when the satellite is outside the volume enclosed by the surface). The plasmapause locations determined from the *in situ* density data (via visual inspection) are indicated by the vertical dashed lines. The intersection locations of the satellite’s orbit to the reconstructed plasmaspheric surface are indicated by the vertical dotted lines. (**b**) Comparison with the electron density data of the VAP-B satellite. Some dashed lines are missed owing to the absence of the corresponding electron density data of VAP-B. Most of the dashed and dotted lines in the right columns of panels a and b are close except for the last ones of VAP-B data due to the complicated local electric density distribution.

In order to verify the reconstructed plasmaspheric configuration based on the EUVC images, we use the *in situ* electron density data of the Electric Field and Waves (EFW)^37^ instruments aboard the two satellites of the Van Allen Probes (VAP) mission^38^ (see Methods for a detailed explanation of the electron density data of VAP) to determine the local positions of the plasmapause during 21 April 2014, and compare it with those deduced from the reconstructed configuration. The result of this comparison is presented in Fig. 5. Figure 5a is for the data of the VAP-A satellite and Fig. 5b is for the VAP-B satellite. The left column of Fig. 5 exhibits the orbits and electron density measurements of the VAP satellites accompanied with the reconstructed configuration in MAG coordinate system. Since the corotation effect of the plasmasphere is eliminated in the MAG coordinate system, the orbit tracks of the two VAP satellites become tortuous and cross in and out of the plasmasphere at different sites of the plasmapause. The right column of Fig. 5 gives the detailed comparison between the locations of the plasmapause deduced from the VAP *in situ* density data and those determined from the reconstructed plasmaspheric configuration along the orbits of VAP. The plasmapause locations obtained from the VAP *in situ* data (via visual inspection; i.e., identifying locations with sharp drops of electron density) are indicated by the vertical dashed lines. The intersection locations of the satellites’ orbit to the plasmapause surface of the reconstructed configuration are indicated by the vertical dotted lines. It is interesting that most of the dashed and dotted lines are close even if the observational times of them are different, which means that the reconstructed plasmaspheric configuration is almost preserved during the observing period. This result verifies the reliability of the reconstructed plasmaspheric configuration based on the EUVC images.

### Relationship between the three plasmaspheric bulges and the substorms

**Figure 6:**
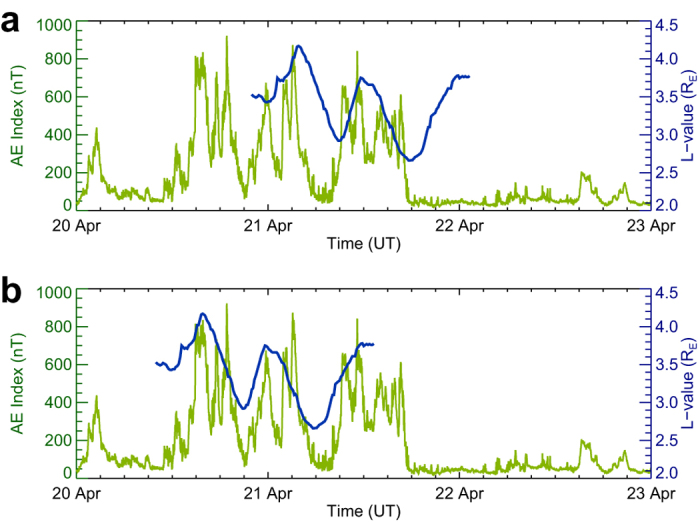
Comparison between the plasmapause positions and the variation of AE index. (**a**) *L*-value curve of the fitting field lines using the midday transit times (blue color) overlying the AE index curve (green color). (**b**) *L*-value curve using the midnight transit times overlying the AE index curve.

The three bulges of the plasmasphere are identified from the dayside shape profiles of the plasmapause, while an examination of the observed EUVC images (see Supplementary Video 1) shows that these bulges might also have appeared previously on the nightside, when three substorms evolved in their expansion phases (see Methods for the approaches to identify the expansion phases). To investigate the phase relationship between the three bulges and the substorms, we deduce the transit times at the midday and midnight MLT (magnetic local time) for all the 153 fitting field lines according to the rotation rate of the Earth (i.e., pure corotation assumption, see Methods for further explanation) and compare them with the variation of the AE index^39^ which acts as proxy of substorm activities^13^. Figure 6a displays the *L*-value curve of the fitting field lines using the midday transit times overlying the AE index curve, and Fig. 6b displays the *L*-value curve shifting back 12 hours (midnight transit time) overlying the AE index curve. It can be seen in Fig. 6 that the deduced time phase of the midnight transits of the three bulges coincides with the time phase of the contemporary substorm activities. This fact implies a causal relationship between the substorms and the bulges of the plasmasphere.

We also compared the midnight transit times of the three bulges with the AL and AU indices^39^, with magnetic bay observations^40^,^41^ from ground-based magnetometers, with the Wp index^42^, and with the SuperMAG auroral electrojet index^43^ (see Methods for details). The result confirms the above conclusion.

## Discussion

From the time series of EUVC images observed during 20–22 April 2014, we identified three bulges which successively appeared at the dayside of the plasmasphere along with the rotation of the Earth. The inferred midnight transit times of the three bulges coincide with the contemporary expansion phases of three substorms. These observations demonstrate that the expansion phases of the three substorms cause the three bulges in the plasmasphere. Different from the concept that geomagnetic storms lead to erosion of the plasmasphere, a substorm can cause plasmaspheric inflation in a local magnetic longitude range around midnight.

A possible mechanism for this inflation process is a rapid filling of the upper magnetic field lines (or flux tubes) at the midnight region during the expansion phase of substorm, which affects the size of the flux tubes. Then these flux tubes rotate to the dayside and are recorded by the EUVC observations. Since the substorms happened successively, the three bulges deduced from EUVC images were also formed successively and thus are distributed at different geomagnetic longitudes. Besides, the newly formed bulge can overlap the previous plasmapause, which leads to a mismatch between the plasmaspheric reconstructions (see the overlapping region in Fig. 4d).

**Figure 7:**
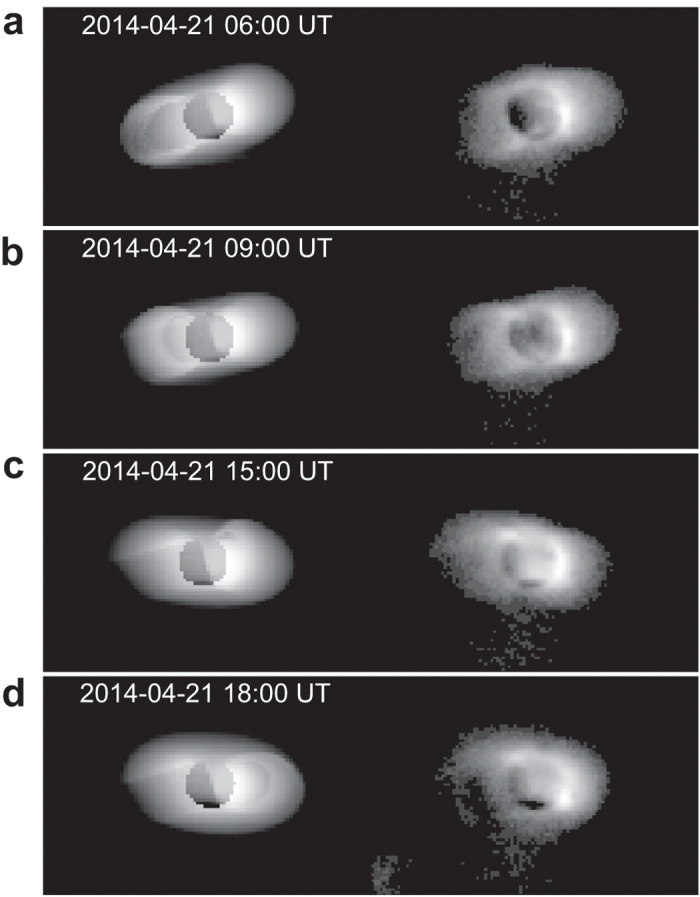
Comparisons between plasmaspheric simulation and observation. (**a**–**d**) Four successive frames of the simulated images which illustrate a filled flux tube (circular-looking plasma loop with larger brightness) corotating from the midnight sector to the midday sector (left) and the corresponding EUVC images (right).

The hypothesis of flux tube rapid filling is inspired by the direct observation of the substorm dynamic process on the nightside of the plasmasphere in the EUVC images around the time period from 8:00 UT to 12:00 UT on 21 April 2014 (see Supplementary Video 1). We performed a simulation of the Moon-based plasmaspheric observations during 21 April 2014 based on the Dynamic Global Core Plasma Model (DGCPM)^3^ to illustrate the suggested mechanism using a rapidly filled flux tube added in the midnight region. This simulation is presented in an animation which is available as Supplementary Video 2 of this paper. Four frames of the simulated images are shown in Fig. 7. The details of the simulation are described in Methods. We will investigate the physical mechanism of the rapid filling process, as well as other kinds of substorm dynamics on the nightside of the plasmasphere (such as cross-L plasmas motion), in future studies.

## Methods

### EUVC imaging principle and data reduction

The EUVC uses a reflective optical system, which includes a spherical multilayer film mirror, a thin film filter, and a spherical photon-counting imaging detector^29^. The optical system is sensitive to the 30.4 nm emission from the Earth’s plasmasphere. The raw data of the EUVC were processed by a series of calibration steps. The data productions include Level 1, Level 2A, and Level 2B data^31^. The Levels 2A and 2B data (in PDS format) are released for scientific research. Compared with the Level 2A data, the Level 2B data add more information in the PDS header, such as geometric positioning parameters^31^. The FOV of the EUVC images in Levels 2A and 2B data is 15° × 15° and the size of the images is 150 × 150 pixels (0.1°/pixel)^31^. The exposure time of each image is 10 min^29^,^31^. The intensity value of each pixel in the images is the number of photons captured by the EUVC during the exposure (dynamic range: 10~10^3^ counts per pixel), which reflects the He^+^ column density of the plasmasphere along LOS^29^,^31^,^32^,^34^.

We perform the data reduction process based on the Level 2B data of EUVC. The data reduction procedure for each EUVC image includes three steps: (1) Rescale the image and let the FOV be 15 × 15 *R*_E_ (i.e., 0.1 *R*_E_/pixel), then the size of Earth radius will be the same (10 pixels/*R*_E_) in all the processed EUVC images (note that in the original EUVC images the apparent radii of the Earth are slightly different owing to the variation of the Earth-Moon distance^44^); (2) Shift the image and place the disk of the Earth at the center of the image^32^,^44^, then the pointing drift effect in the original EUVC images is eliminated; (3) Rotate the image and place the projected geomagnetic dipole axis along the y-axis direction^32^, which is convenient for the profile fitting using dipole field lines. The observational parameters needed for the data reduction process are available in the header of the Level 2B data. A demo of the data reduction procedure can be found in ref. 32. All the 153 processed EUVC images during the observing period (20–22 April 2014) are combined into an animation which is available as Supplementary Video 1 to this paper.

The noise in the EUVC images shown in Supplementary Video 1 comes from the contamination by sunlight^31^. The higher noise level at the beginning of the animation is owing to the higher elevation angle of the Sun^31^.

### Geophysical coordinate systems

The definitions of the geophysical coordinate systems^45^,^46^ used in this paper are given in Table 1. The coordinate transformations were conducted using the IDL Geopack DLM (Dynamic Link Module), which is based on the original GEOPACK Fortran library.

### Orbit of the Moon

The orbit positions of the Moon in the whole month of April 2014 shown in Fig. 1 are computed using the code (moonpos.pro) provided by the NASA’s IDL Astronomy Library^47^.

### Geomagnetic field model

We use the dipole field model to calculate the geomagnetic field lines. The dipole axis is defined by the International Geomagnetic Reference Field (IGRF) model^48^,^49^. The practical tracing of the field lines is conducted using the IDL Geopack DLM.

The *L*-values of the plasmapause locations studied in this paper are less than 4.2 *R*_E_ (see Fig. 4a) and we only measure the dayside plasmapause positions which are less affected by the stretching effect of field lines during substorms. Thus we adopt the dipole field model for the plasmapause fitting in this work. When investigating the nightside plasmapause dynamics during the substorms in the future studies, we will use more sophisticated geomagnetic field models, such as the TS07D model^50^,^51^.

### *In situ* electron density data of VAP

Two instruments aboard the VAP satellites can provide *in situ* electron density measurements. One instrument is the Electric and Magnetic Field Instrument Suite and Integrated Science (EMFISIS)^52^ which determines the electron density by tracking the upper hybrid frequency^53^. Another instrument is the Electric Field and Waves (EFW) which estimates the electron density by tracking the spacecraft potential^37^. The electron density value of EMFISIS is more accurate than that of EFW^53^ (note that EFW relies on the values of EMFISIS to calibrate its results^37^,^53^), but the EFW measurements have higher time resolution and time coverage^37^.

During the observing period of the EUVC images (20–22 April 2014), only the EFW electron density data are available. So we adopt the EFW electron density measurements in this paper (see electron density plots in Fig. 5). The *in situ* electron density values (as well as the timing and orbit data of VAP satellites) are taken from the Level 3 data files (in CDF format) of the EFW. Although the EFW data is not as accurate as the EMFISIS data, they are sufficient for determining the plasmapause positions via visual inspection (i.e., identifying positions with sharp drops of electron density; see the curves of electron density in Fig. 5). Figure 5 displays only the reliable electron density data (according to the reliability flags contained in the CDF data files). The gaps in the curves correspond to unreliable or missing data.

### Comparison of the three plasmaspheric bulges with substorm observations

**Figure 8:**
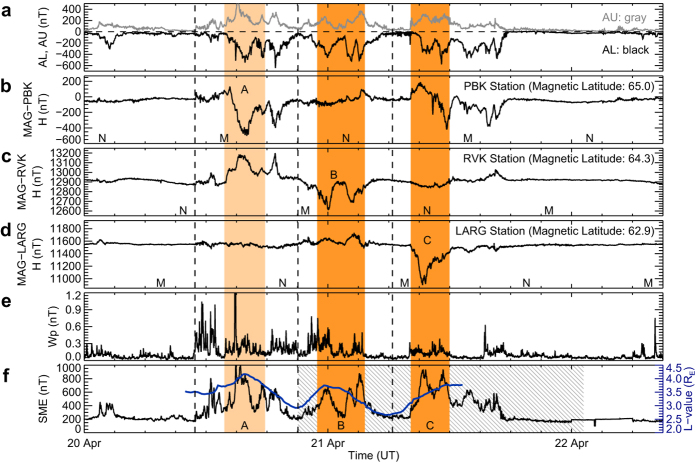
Comparison of the midnight transit times of the three bulges with various substorm observations. (**a**) AL (negative; black color) and AU (positive; gray color) indices. (**b**) H-component (along north direction) recorded by the magnetometer at PBK. The magnetic midnight and noon UT times of the station are marked with the letters “M” and “N” on the time axis, respectively (the same below). (**c**) H-component at RVK. (**d**) H-component at LARG. (**e**) Wp Index. (**f**) SuperMAG auroral electrojet index (SME index; black color). The *L*-value curve (blue color) of three plasmaspheric bulges using the midnight transit times is plotted overlying the SME curve for comparison. The hatched area indicates the EUVC observing period. The three red shaded areas represent the expansion phases of the three substorms (labeled A, B, and C, respectively).

In Fig. 8, we compare the midnight transit times of the three plasmaspheric bulges with various substorm observations, which include the time series curves of the AL and AU indices^39^ (Fig. 8a), magnetic bay observations^40^,^41^ from three ground-based stations PBK (geographic latitude 70.9°, longitude 170.9°; magnetic latitude 65.0°; Fig. 8b), RVK (geographic latitude 65.0°, longitude 11.0°; magnetic latitude 64.3°; Fig. 8c), and LARG (geographic latitude 55.2°, longitude 254.7°; magnetic latitude 62.9°; Fig. 8d), the Wp index^42^ (Fig. 8e), and the SuperMAG auroral electrojet index^43^ (SME index; Fig. 8f). The *L*-value curve of the three bulges using the midnight transit times (the same *L*-value curve as in Fig. 6b) is plotted overlying the SME curve in Fig. 8f for comparison.

In Fig. 8, the three substorms corresponding to the three bulges (labeled A, B, and C, respectively, as in Fig. 4) are separated by three vertical dashed lines according to the minimum values of the SME index curve (see Fig. 8f). The occurrence times of substorms B and C are covered by the observing period of the EUVC images (indicated by the hatched area in Fig. 8f), and the expansion phases of the substorms B and C can be directly identified from the EUVC images by the intensification of foot points of the plasma loops around midnight region. The deduced expansion phases of the substorms B and C are highlighted with two deep-red shaded areas in Fig. 8 (from 2014-04-20 22:56 UT to 2014-04-21 03:38 UT for substorm B and from 2014-04-21 08:09 UT to 2014-04-21 11:58 UT for substorm C). It can be seen in Fig. 8f that the expansion phases of substorms B and C coincide with the midnight transit times of the bulges B and C.

The occurrence time of substorm A is not covered by the EUVC observing period (see the hatched area in Fig. 8f), so we assume that its probable expansion phase is centered at the peak position of the *L*-value curve associated with bulge A (see Fig. 8f) and the duration of the expansion phase is four hours (referring to the observed durations of the substorms B and C). The inferred probable expansion phase of substorm A is indicated by a light-red shaded area (centered at 2014-04-20 15:48 UT) in Fig. 8.

Figure 8b–d give the three magnetic bay observations for substorms A, B, and C, respectively. It can be seen that the expansion phases of the three substorms (represented by the three red shaded areas) coincide with the three magnetic bays which are marked with the capital letters A, B, and C in Fig. 8b–d, respectively. The Wp index curve in Fig. 8e represents the signal of mid-latitude Pi2 pulsations^54^ for substorm onset time identification^42^, which also shows compatibility with the derived expansion phases of the three substorms.

The AE indices (including AL and AU indices) data are supplied by World Data Center for Geomagnetism, Kyoto. The ground-based magnetometer data are provided by NASA’s Coordinated Data Analysis Web (CDAWeb). The Wp index data are supplied by the Substorm Swift Search (S^3^, S-cubed) web site (http://s-cubed.info). The SuperMAG auroral electrojet index data are supplied by the SuperMAG project (http://supermag.jhuapl.edu/indices).

### Pure corotation assumption for the three plasmaspheric bulges

The plasmasphere as a whole corotates with the Earth^1^. Higher geomagnetic activity and local-time-dependent convection may lag the rotation rates of certain plasmaspheric features^55^. In this paper we adopt the pure corotation assumption to calculate the midday and midnight transit times of the three bulges because: (1) During the observing period of the EUVC images (20–22 April 2014), the geomagnetic activity is relatively low (*Kp* ~ 4) and then the corotation of the plasmasphere is less disturbed; (2) The bulges are large-scale plasmaspheric structure, thus they are relatively stable and are less affected by local convection (see Supplementary Video 1 for an intuitive impression).

### Simulation of Moon-based plasmaspheric observations

The simulation is based on the DGCPM model^3^. An empirical model on the field-aligned plasmaspheric density distribution^7^ is used to get the off-equatorial densities from DGCPM. The density in the Earth’s shadow is set to zero. The simulated images are in log scale. A flux tube is added in the premidnight sector (assuming that the flux tube fills at high rate for a short period of time), which produces a circular-looking plasma loop with larger brightness in the images. This simulation is presented in an animation (Supplementary Video 2). Four frames of the simulated images are shown in Fig. 7.

## Additional Information

**How to cite this article**: He, H. *et al*. Response of plasmaspheric configuration to substorms revealed by Chang’e 3. *Sci. Rep.* **6**, 32362; doi: 10.1038/srep32362 (2016).

## Supplementary Material

Supplementary Video 1

Supplementary Video 2

Supplementary Information

## Figures and Tables

**Table 1:** Definitions of the geophysical coordinate systems.

Coordinate system	Axis	Definition
Geocentric Equatorial Inertial (GEI)	X_GEI_	Pointing from the Earth towards the position of the Sun at the vernal equinox (intersection of the Earth’s equatorial plane and the ecliptic plane)
	Z_GEI_	Parallel to the Earth’s rotation axis (north is positive)
	Y_GEI_	Z_GEI_ × X_GEI_
Geomagnetic (MAG)	Z_MAG_	Parallel to the magnetic dipole axis (north is positive)
	Y_MAG_	Z_GEI_ × Z_MAG_
	X_MAG_	Y_MAG_ × Z_MAG_
Geocentric Solar Magnetospheric (GSM)	X_GSM_	Pointing from the Earth to the Sun
	Y_GSM_	Z_MAG_ × X_GSM_
	Z_GSM_	X_GSM_ × Y_GSM_
Solar Magnetic (SM)	Z_SM_	Parallel to the magnetic dipole axis (north is positive; same as Z_MAG_)
	Y_SM_	Z_SM_ × X_GSM_ (same as Y_GSM_)
	X_SM_	Y_SM_ × Z_SM_

Note that the definitions give the directions of the axes instead of the unit vectors. The GSM coordinate system is not used in this paper but is referred to by the definition of the SM coordinate system^45^.
