# Supplementary material for: Response of plasmaspheric configuration to substorms revealed by Chang’e 3
Source: Sci Rep. 2016 Aug 31;6:32362. doi: 10.1038/srep32362 (PMC5006020; doi:10.1038/srep32362)
Supplement: Supplementary Information [file srep32362-s3.pdf]

Response of plasmaspheric configuration to substorms revealed by Chang'e 3

Han He*[1,2], Chao Shen*[3,4], Huaning Wang*[1,2], Xiaoxin Zhang[5], Bo Chen[6], Jun Yan[1], Yongliao Zou[1], Anders M. Jorgensen[7], Fei He[6], Yan Yan[1,2], Xiaoshuai Zhu[1,2], Ya Huang[4] & Ronglan Xu[4]

[1] National Astronomical Observatories, Chinese Academy of Sciences, Beijing, China.
[2] Key Laboratory of Solar Activity, Chinese Academy of Sciences, Beijing, China.
[3] School of Natural Sciences and Humanity, Harbin Institute of Technology Shenzhen Graduate School, Shenzhen, China. [4] State Key Laboratory of Space Weather and National Space Science Center, Chinese Academy of Sciences, Beijing, China.
[5] National Center for Space Weather, China Meteorological Administration, Beijing, China. [6] Changchun Institute of Optics, Fine Mechanics and Physics, Chinese Academy of Sciences, Changchun, China. [7] Electrical Engineering Department, New Mexico Institute of Mining and Technology, Socorro, New Mexico, USA.

* Correspondence and requests for materials should be addressed to
H.H. (hehan@nao.cas.cn) or C.S. (sc@nssc.ac.cn) or H.N.W. (hnwang@nao.cas.cn)

Supplementary Video 1. Animation of the 153 frames of the processed EUVC images observed during 20-22 April 2014. The images are displayed in log scale. The noise in the EUVC images shown in the animation comes from the contamination by sunlight. The higher noise level at the beginning of the animation is owing to the higher elevation angle of the Sun. The video file is in MOV format.

Supplementary Video 2. Animation to present a simulation of the Moon-based plasmaspheric observations during 21 April 2014. This simulation illustrates a filled flux tube (circular-looking plasma loop with larger brightness) corotating from the midnight sector to the midday sector. Both the simulation images (left) and the corresponding EUVC images (right) are shown in the animation. The images are in log scale. The video file is in MOV format.
